# Rapid and Visual Detection of Porcine Parvovirus Using an ERA-CRISPR/Cas12a System Combined With Lateral Flow Dipstick Assay

**DOI:** 10.3389/fcimb.2022.879887

**Published:** 2022-05-11

**Authors:** Jing Wei, Yanan Li, Yingli Cao, Qi Liu, Kankan Yang, Xiangjun Song, Ying Shao, Kezong Qi, Jian Tu

**Affiliations:** ^1^ Anhui Province Engineering Laboratory for Animal Food Quality and Bio-safety, College of Animal Science and Technology, Anhui Agricultural University, Hefei, China; ^2^ Anhui Province Key Laboratory of Veterinary Pathobiology and Disease Control, Anhui Agricultural University, Hefei, China

**Keywords:** CRISPR-Cas12a, enzymatic recombinase amplification, porcine parvovirus, lateral flow dipstick, rapid detection

## Abstract

Porcine parvovirus (PPV) is one of the important causes of pig reproductive diseases. The most prevalent methods for PPV authentication are the polymerase chain reaction (PCR), enzyme-linked immunosorbent assay, and quantitative real-time PCR. However, these procedures have downsides, such as the fact that they take a long time and require expensive equipment. As a result, a rapid, visible, and economical clinical diagnostic strategy to detect PPV is necessary. In this study, three pairs of crRNA primers were designed to recognize the VP2 gene, and an ERA-CRISPR/Cas12a system for PPV detection was successfully developed. The approach involved isothermal detection at 37°C, and the method can be used for visual inspection. The detection limit of the ERA-CRISPR/Cas12a system was 3.75 × 10^2^ copies/μL, and no cross reactions with other porcine viruses were found. In view of the preceding, a rapid, visible, and low-cost nucleic acid testing approach for PPV has been developed using the ERA-CRISPR/Cas12a system.

## Introduction

Porcine parvovirus belongs to the *Parvoviridae family*, which consists of two subfamilies *Parvovirinae* and *Densovirinae*. It is a leading cause of reproductive failure in pigs, which is a serious issue in the pig breeding industry. Amto Mayr and colleagues in Munich, Germany, used primary pig cells to propagate the classical swine fever virus in 1965, and discovered and isolated compromised porcine parvovirus (PPV), proving the presence of the virus. Seven parvovirus species, PPV1–7, have been discovered in pigs so far. PPV1 is the dominant causal agent in swine herds, and it was initially found in Germany in 1965 (Streck and Truyen, 2020). The incidence of infections with PPV2–7 is low and the clinical signs are not obvious. PPV1 infection gives rise to embryonic mortality, infertility, stillbirths and other signs; involvement of the other porcine parvovirus serotypes in reproductive failure is still to be determined (Ellis et al., 2000; Kennedy et al., 2000).

PPV1 is a hexagonal, circular or symmetrical icosahedral DNA virus with a diameter between 22 and 23 nm and has no capsule. The genome of PPV1 mainly consists of two open reading frames (ORFs), ORF1 encoding non-structural proteins NS1, NS2 and NS3; and ORF2 encoding structural proteins VP1 and VP2. VP2 accounts for more than 90% of the capsid components of the virus, and the VP2 gene is the most important structural protein and immunogenic protein of PPV. The sequence homology of VP2 genes of different genotypes ranges from 26.5% to 40.0%. It not only stimulates the immune response of the host but also plays an important role in the replication process of the virus (Kamstrup et al., 1998; Streck et al., 2011; Xu et al., 2013). PPV1 can occur in different seasons, and pigs of different varieties, sexes, purposes and ages, as well as wild boar, can be infected. The virus mainly causes signs in pregnant sows, especially primiparous sows, resulting in sick pigs, abortion, stillbirth, fetal deformities, and mummies. Repeated infections are associated with conditions such as infertility, which affects the sow’s farrowing rate, and thus affects the economic benefits of pig breeding farms, causing serious economic losses (Meszaros et al., 2017).

At present, the clinical detection of PPV is based mainly on polymerase chain reaction (PCR), enzyme-linked immunosorbent assay (ELISA), real-time PCR, etc. PCR is the main method used to detect parvovirus (Huang et al., 2004; Caprioli et al., 2006; Jiang et al., 2010), but it is insufficiently sensitive and the amplification efficiency may be compromised by many factors (Green and Sambrook, 2018). ELISA is also commonly used to detect parvovirus (Deng et al., 2018), but its sample processing procedures are relatively complex and time-consuming, and easily produce false positive (Terato et al., 2016). Both real-time PCR and loop-mediated isothermal amplification (LAMP) are also used for detection of PPV and, although they are highly sensitive, they require expensive equipment and are time-consuming, limiting their use in daily testing and rapid field detection (H.-Y. Chen et al., 2009; H.-t. Chen et al., 2010).

Therefore, a convenient and simple approach for identifying PPV was essential. Enzymatic recombinase amplification (ERA) is an upgrade of recombinase polymerase amplification (RPA) and can be operated at constant temperature without thermal cycling. ERA achieves rapid amplification of nucleic acid by the simultaneous action of multiple functional proteins at constant temperature, with strong specificity, high sensitivity and easy operation (Yu et al., 2015; Mukama et al., 2020). With the continuous updating of rapid molecular diagnostic technology, nucleic acid detection technology has been developed and applied that is based on the CRISPR-Cas system, a short palindrome repeat sequence with regular aggregation in the prokaryotic immune system. Studies have shown that several endonucleases in the CRISPR-Cas system (Cas12a/b, Cas13a/b and Cas14) are characterized by lateral cleavage activity, and Cas12a is capable of nonspecifically cleaving single-stranded DNA and binding to the corresponding target site. The CRISPR-Cas system, a nucleic acid detection tool, shows great potential in establishing novel molecular diagnostic methods, owing to its reliability, high specificity, and sensitivity (Guk et al., 2017; S.-Y. Li et al., 2018; L. Li et al., 2019; B. Wang et al., 2019).

In this study, we established a molecular detection system aimed at the VP2 gene for PPV detection that combined ERA-CRISPR/Cas12a technology and lateral flow dipstick approaches. The method had the advantages of rapidity and high specificity, it was easy to read and had no professional operation requirement.

## Materials and Methods

### Sample Source and Nucleic Acid Extraction

**Figure 1:**
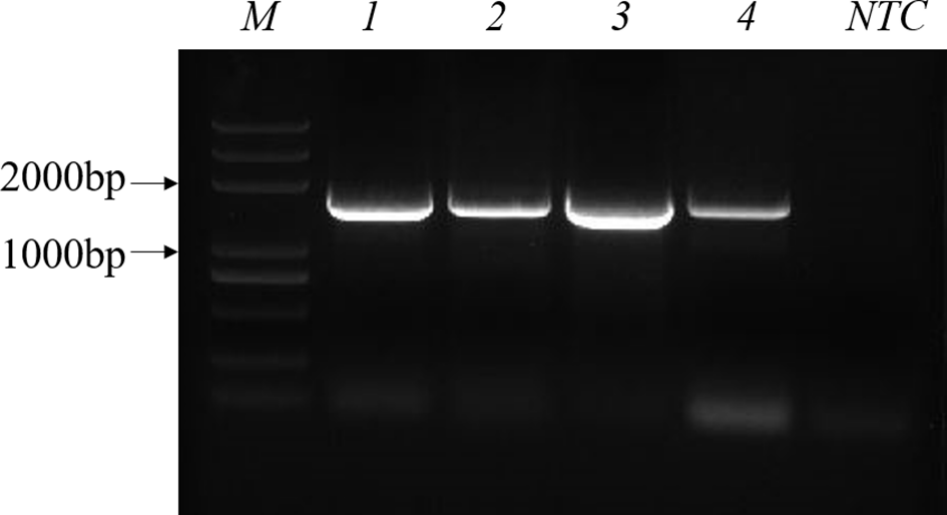
Results of PCR agarose gel electrophoresis. M: Marker; 1–4: PPV-VP2 plasmid; NTC: negative control with ddH_2_O.

PPV2, porcine circovirus type 3 (PCV3) and PPV were stored in the Anhui Province Key Laboratory of Veterinary Pathobiology and Disease Control. Porcine pseudorabies live vaccine (PRV, HB-98 strain), porcine epidemic diarrhea live vaccine (PEDV, ZJ08 strain), swine fever live vaccine (CSFV, AV1412 strain), and porcine reproductive and respiratory syndrome live vaccine (PRRSV, R98 strain) were purchased from China Animal Husbandry Industry Co., LTD. Clinical materials, including the liver, spleen and kidney, were collected from pig farms suspected to be infected with PPV in Anhui Province, and donated by the Anhui Animal Disease Prevention and Control Center. Viral DNA was extracted from diseased tissues using a commercial viral genome DNA/RNA extraction kit, the TIANamp Virus DNA/RNA Kit (TIANGEN, Beijing, China), for immediate use or storage at −20°C. Plasmids were extracted according to the instructions for the SanPrep Column Plasmid Mini-Preps Kit (Sangon Biotech, Shanghai, China). Agarose gel electrophoresis was performed on the extracted plasmids, as shown in **Figure 1**.

### Design of Primers and Probes

**Figure 2:**
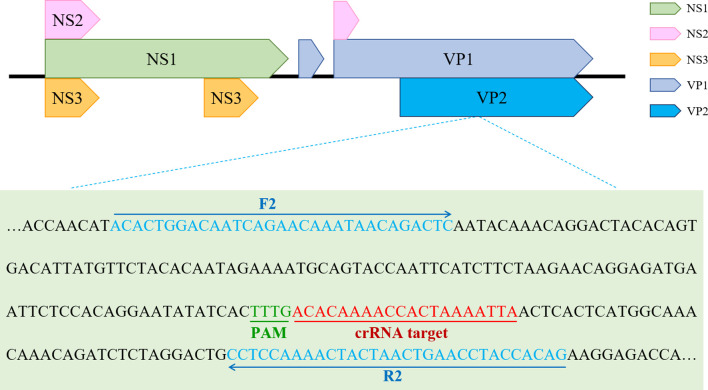
The optimal primer pairs for ERA and crRNA binding sites in the target VP2 gene sequence of PPV.

The primers used for ERA-CRISPR/Cas12a detection were designed to be within the conserved region of PPV, encoding structural protein VP2, according to the crRNA primer principle of CRISPR/Cas12a, as shown in **Figure 2**. According to the design principles for crRNA primers, three pairs of crRNA primers were designed using the online CRISPR primer design websites of Liang Cpf1 (http://bioinfolab.miamioh.edu/CRISPR-DT/). According to the principles of ERA primer design, three upstream and three downstream primers were designed using Primer Premier 5 for the conserved sequence of the VP2 gene. Primers and probes were synthesized by Anhui General Co., LTD. (Anhui, China). Oligonucleotide sequences of the CRISPR/Cas12a primers and probes used in this study are shown in **Table 1**.

**Table 1:** Sequences of the primers and LFD probe used in this work.

Primers	Sequences (5’-3’)	Size/bp
VP2-F	GTCTGCAACAGGAAATGAATC	1658
VP2-R	ATTCTGGAAACATTCTTATGC	
qPCR-F	GGGGAGGGCTTGGTTAGAAT	319
qPCR-R	TTGGTGGTGAGGTTGCTGAT	
ERA-1-F	ACACTGGACAATCAGAACAAATAACAGACTC	227
ERA-1-R	CTGTGGTAGGTTCAGTTAGTAGTTTTGGAGG	
ERA-2-F	ACCAACATACACTGGACAATCAGAACAAATA	225
ERA-2-R	GGTAGGTTCAGTTAGTAGTTTTGGAGGCAGT	
ERA-3-F	ATGCAGTACCAATTCATCTTCTAAGAACAGGA	147
ERA-3-R	TGGTAGGTTCAGTTAGTAGTTTTGGAGGCAGT	
crRNA-1-F	GAAATTAATACGACTCACTATAGGGTAATTTCTACTAAGTGTAGATTAAAAATAGCACCAAACCTA	66
crRNA-1-R	TAGGTTTGGTGCTATTTTTAATCTACACTTAGTAGAAATTACCCTATAGTGAGTCGTATTAATTTC	
crRNA-2-F	GAAATTAATACGACTCACTATAGGGTAATTTCTACTAAGTGTAGATACACAAAACCACTAAAATTA	66
crRNA-2-R	TAATTTTAGTGGTTTTGTGTATCTACACTTAGTAGAAATTACCCTATAGTGAGTCGTATTAATTTC	
crRNA-3-F	GAAATTAATACGACTCACTATAGGGTAATTTCTACTAAGTGTAGATTAAAAACAATCCACCAGGAC	66
crRNA-3-R	GTCCTGGTGGATTGTTTTTAATCTACACTTAGTAGAAATTACCCTATAGTGAGTCGTATTAATTTC	
Probe	6-FAM-TTTTTT-BHQ1	
	6-FAM-TTTTTTTATTTTTTT-Biotin	

### The ERA-CRISPR/Cas12a/LFD Assay

**Figure 3:**
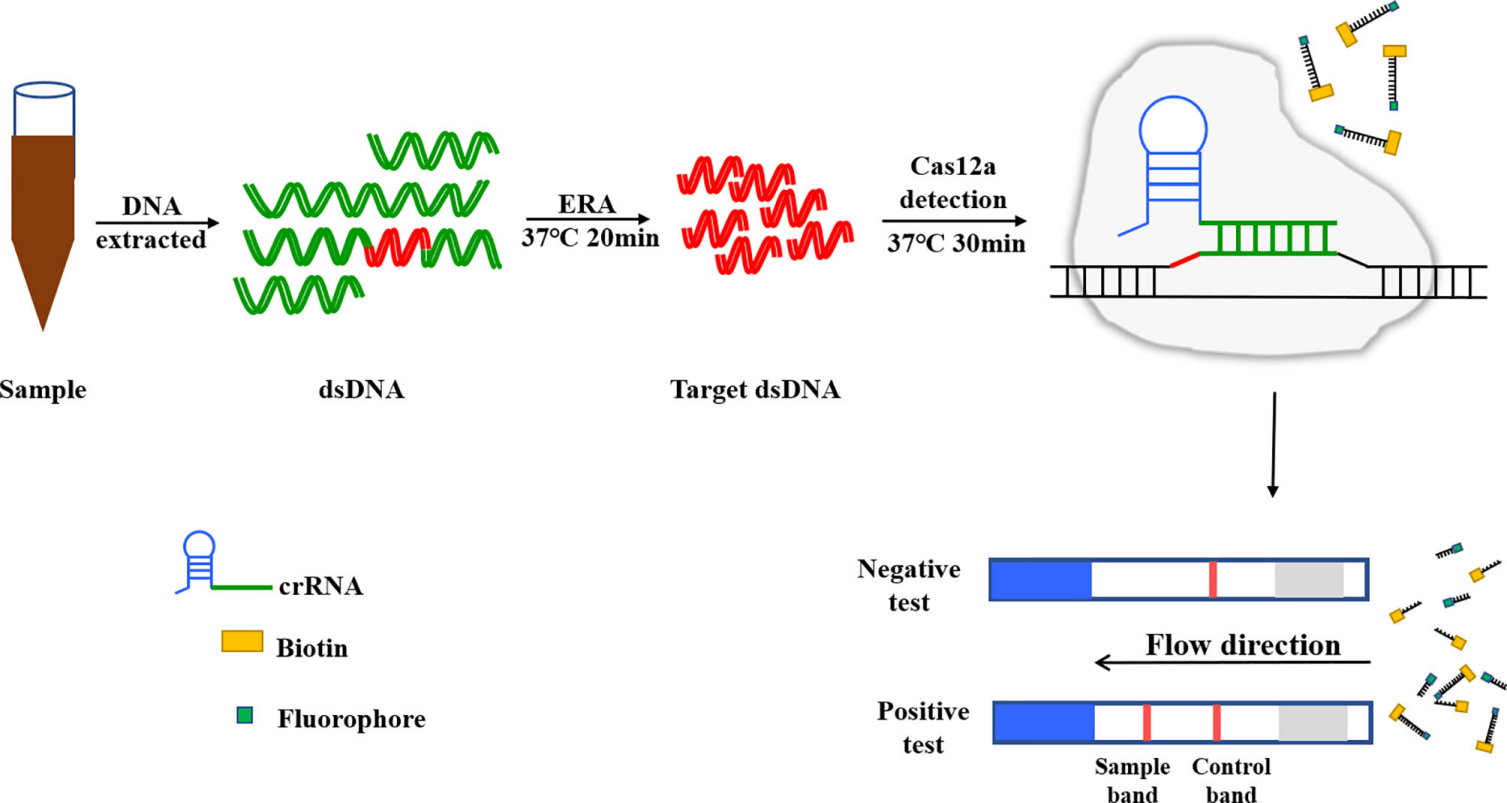
CRISPR/Cas12a based lateral flow detection, experimental workflow.

**Figure 4:**
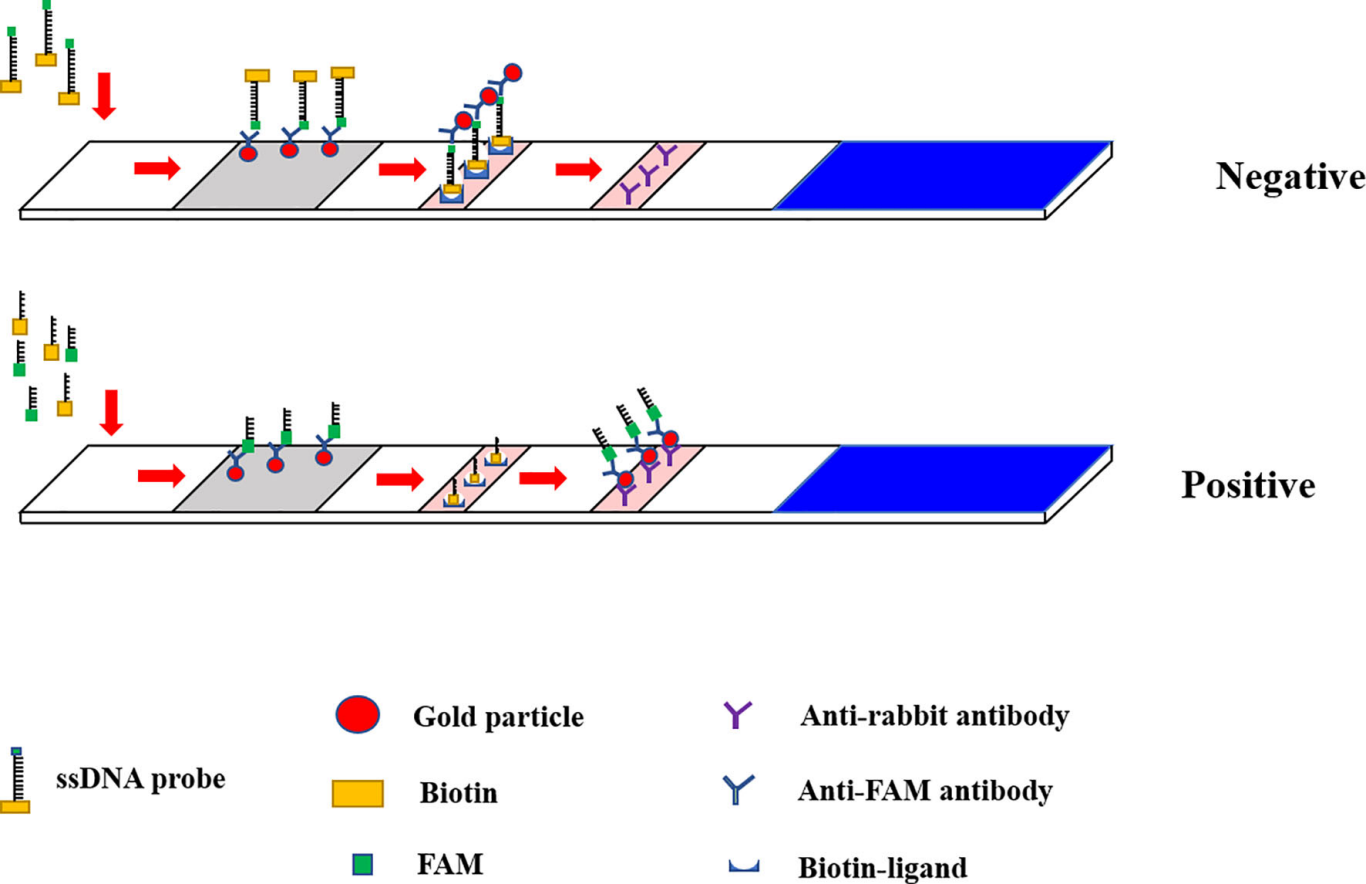
Schematic diagram of Lateral flow dipstick detection.

Lateral flow dipsticks were purchased from Tiosbio (Beijing, China), and EnGenVR Lba Cas12a (Cpf1) (M0653T) and NEBuffer 2.1 (B7203S) were purchased from New England Biolabs (NEB). Mouse RNase inhibitors were purchased from Vazyme Biotech Co., Ltd. (R301-01). The total reaction system of CRISPR/Cas12a/LFD was made up to 20 μL, and consisted of 2 μL NEBuffer 2.1, 1 μL RNase inhibitor, 1 μL 500 nM crRNA, 0.5 μL Cas12a, 0.6 μL probe, 6 μL plasmid template and 8.9 μL ddH_2_O. The reaction system was added to the Eppendorf, gently blown and mixed, oscillated and centrifuged for 1 s. The reaction mixture was incubated in a water bath for 30 min at 39°C. After the reaction, the reaction tube was opened and the system replenished with ultra-pure water to 50 μL, blown and mixed well. The binding pad end of the test strip was inserted into the reaction tube, with the liquid level not above the top of the binding pad **(** **Figure 3** **)**. After the Absorbent Pad was fully infiltrated and the quality control line (C line) was colored, the strip was removed. Red strips on both the test line and the quality control line indicated a positive result, while only the quality control line being red indicated a negative result. Only one test line showed suspicious results and needed to be retested **(** **Figure 4** **)**.

### Sensitivity and Specificity of the Assay

The recombinant plasmid pMD-19T-VP2 was constructed by ligating the pMD-19T Vector (TaKaRa) with the conserved sequence of the VP2 gene for 30 min at 16°C, and was transformed into DH5α receptor cells (Tsingke, Nanjing, China). The recombinant plasmid was sequenced by Nanjing Tsingke Biotechnology Co., LTD. The plasmid concentration was determined with an ND-2000c spectrophotometer (NanoDrop, Wilmington, DE, USA), and the plasmid copy number was calculated according to the formula: number of copies = (amount × 6.02 × 10^23^)/(length × 10^9^ × 660). The plasmid was diluted to 10^6^–10^1^ copies/μL and stored at −20°C. PPV2, PCV3, PRV (HB-98 strain), CSFV (AV1412 strain), PEDV (ZJ08 strain) and PRRSV (R98 strain) specific tests were performed.

### Evaluation of the ERA-CRISPR/Cas12a/LFD Assay Using Clinical Samples

The ERA-CRISPR/Cas12a/LFD was performed on 15 tissue samples to evaluate the specificity of the assay. Nucleic acids in clinical samples were amplified by the ERA method: the reaction system (50 μL) contained Lytic Agent, 20 μL; forward primer (10 μM), 2.5 μL; reverse primer (10 μM), 2.5 μL; template and water, 23 μL. The mixture was placed in the tube, mixed thoroughly and centrifuged briefly after which 2 μL activator was added to the reaction cap, the tube cap was closed, and centrifuge briefly. The amplified samples were tested by CRISPR/Cas12a combined with the lateral flow dipstick, and the reaction results were compared with qPCR.

## Results

### Design and Screening of the ERA Primers

**Figure 5:**
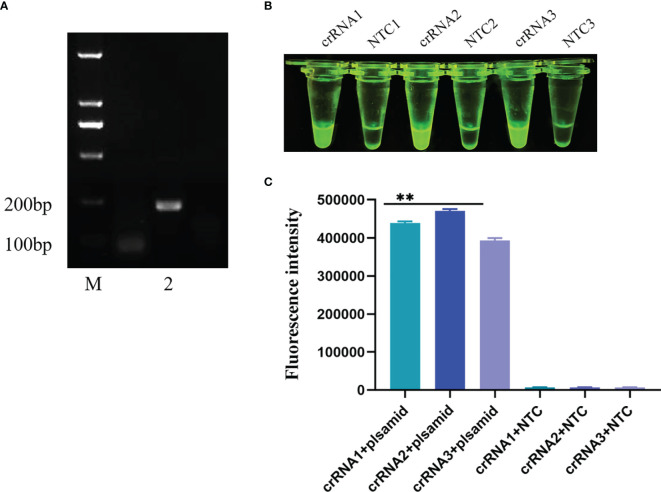
Screening of crRNA primers. **(A)** ERA primer was applied to agarose gel electrophoresis after nucleic acid amplification. M: Marker; 2: The second upstream primer and the second downstream primer constitute the ERA primers for nucleic acid amplification results. **(B, C)** Three pairs of crRNA primers visualized for CRISPR/Cas12a. Comparison of results and fluorescence values under UV light. All data are presented as the statistical significance of differences were **p < 0.01.

Three pairs of ERA upstream primers were paired with three pairs of downstream primers, and the upstream primers were utilized to screen the downstream primers, while the upstream primers were screened by the chosen downstream primers. To evaluate the nine primer pairs for ERA, the ERA reaction was carried out for 20 min at 39°C, and the resultant products were evaluated on an electrophoresis gel. After the nucleic acid of clinical samples was amplified by these nine pairs of primers and agarose nucleic acid gel electrophoresis was performed, the results revealed that the second pair of primers had the best efficiency. The primers and probes detected by ERA-CRISPR/Cas12a were created artificially, as shown in **Figure 5A**.

### Optimization of the crRNA Primers

The CRISPR/Cas12a visualization test was performed after three pairs of crRNA primers were purified. The reaction was carried out in a water bath at 39°C for 30 minutes, the fluorescence values were measured on ABI StepOnePlusTM (Applied Biosystems) equipment after the reaction. All three pairs of primers were able to amplify the target band, as shown in **Figures 5B, C**, but the second pair was the brightest and had the highest fluorescence value under UV light. The second pair had the greatest effect on crRNA primers, therefore it was utilized in the following tests.

### Comparing the Sensitivity of ERA-CRISPR/Cas12a/LFD and PCR Amplification

**Figure 6:**
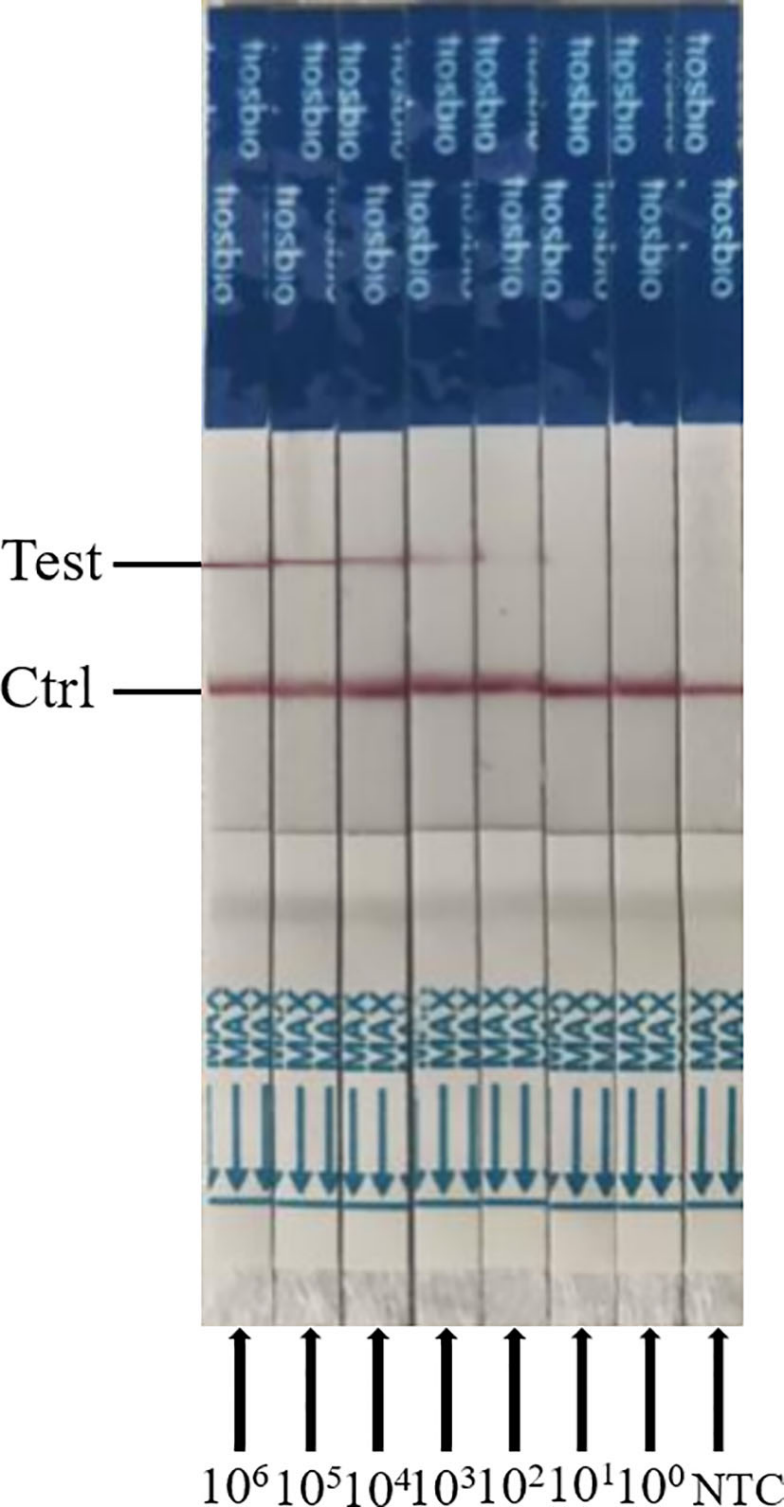
Analytical sensitivity results of ERA-CRISPR/Cas12a/LFD for the plasmid; 10^6^ to 10^0^ indicate tests with 3.75 × 10^6^ to 3.75 copies/μL recombinant plasmid as a template. NTC indicates a test with RNase-free ddH_2_O as a template.

To evaluate the detection sensitivity of ERA-CRISPR/Cas12a/LFD, the plasmid pMD-19T-VP2 was diluted from 3.75 × 10^6^ to 3.75 × 10^0^ copies/μL, and was used as a template. The detection sensitivity of the PCR and ERA-CRISPR/Cas12a/LFD assays was compared. We found that the limit of detection for the general PCR assay for the PPV VP2 gene was only 3.75 × 10^4^ copies (data not shown). As shown in **Figure 6**, the detection sensitivity of ERA-CRISPR/Cas12a/LFD for the PPV VP2 gene was 3.75 × 10^2^ copies. The experimental results showed that the ERA-CRISPR/Cas12a/LFD assay was more sensitive than the conventional PCR assay in detecting PPV.

### Specificity Assessment of ERA-CRISPR/Cas12a/LFD Assay

**Figure 7:**
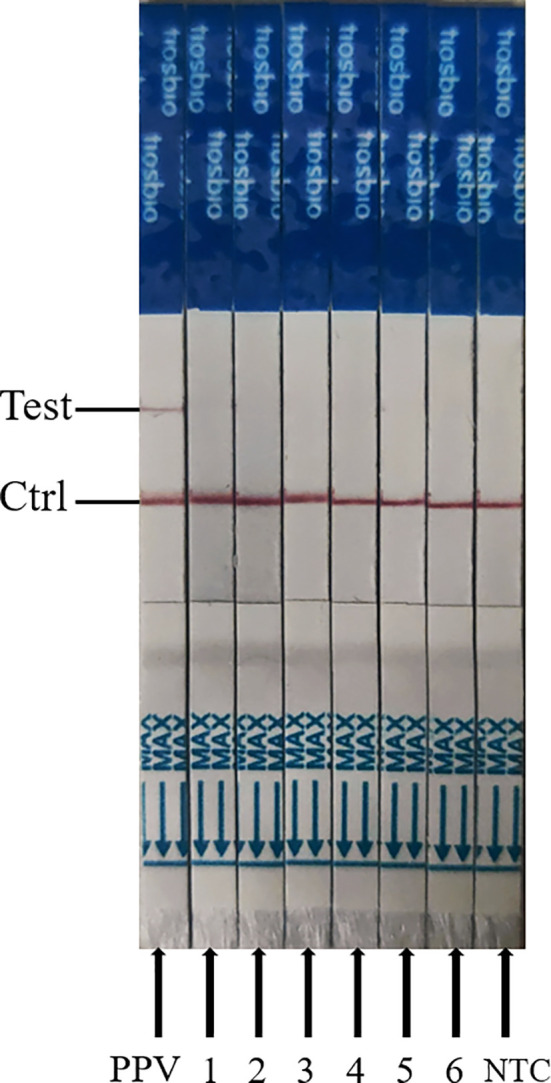
Analytical specificity result of the ERA-CRISPR/Cas12a assay. From left to right, the test samples are positive samples of PPV; 1, PPV-2; 2, PCV3; 3, porcine pseudorabies live vaccine (PRV, HB-98 strain); 4, swine fever live vaccine (CSFV, AV1412 strain); 5, porcine epidemic diarrhea live vaccine (PEDV, ZJ08 strain); 6, porcine reproductive and respiratory syndrome live vaccine (PRRSV, R98 strain); NTC, negative control of RNase-free ddH_2_O.

To appraise the detection specificity of ERA-CRISPR/Cas12a/LFD, the genomic DNA or cDNA from PPV2, PCV3, PRV, CAFV, PEDV, and PRRSV was used. As shown in **Figure 7**, we discovered that only the PPV1 sample was positive, with bands at both the detection and quality control lines, while the other virus samples and negative control were negative (only the quality control line had bands). These results suggested that ERA-CRISPR/Cas12a/LFD could effectively distinguish PPV from other viruses. The ERA-CRISPR/Cas12a/LFD showed high specificity for PPV detection (100%).

### Performance of ERA-CRISPR/Cas12a/LFD on Clinical Samples Tested for PPV

**Figure 8:**
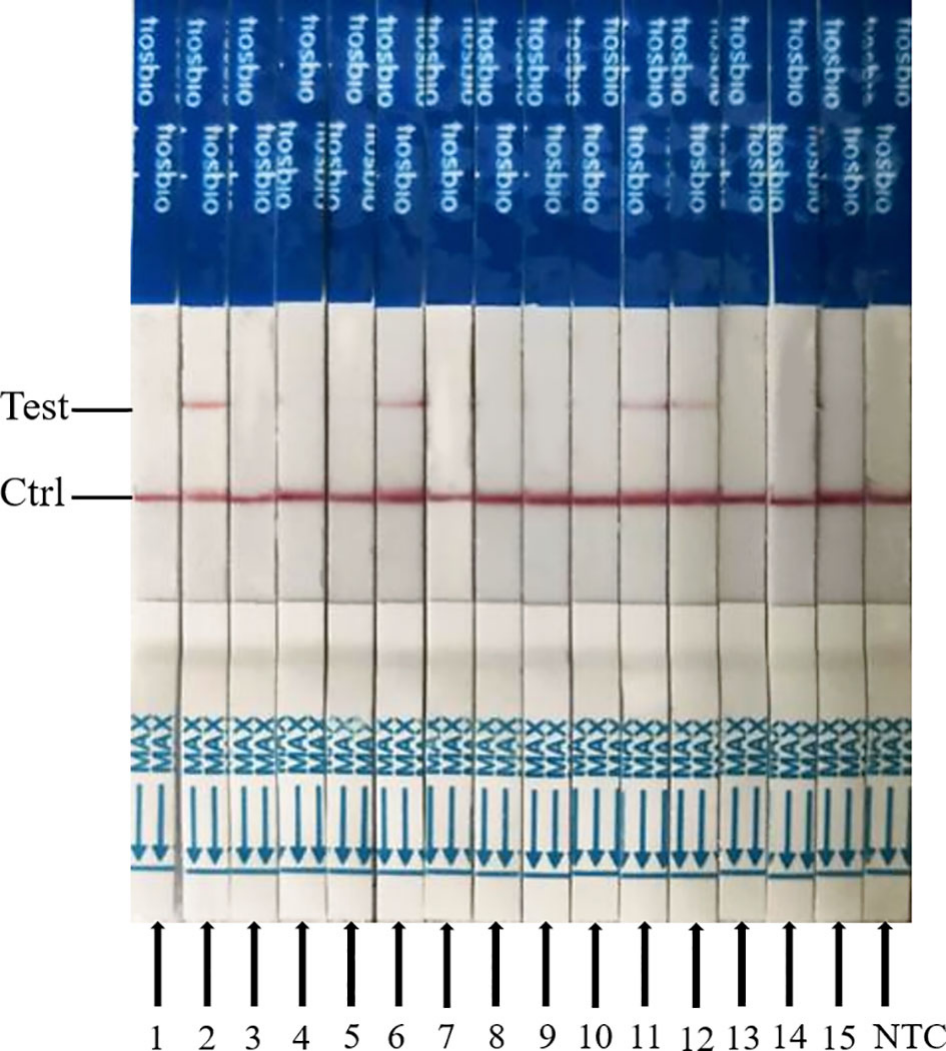
Clinical sample results of ERA-CRISPR/Cas12a combined with ERA analysis. 1–15 indicate different clinical samples; NTC, negative control of RNase-free ddH_2_O.

The ERA-CRISPR/Cas12a/LFD and qPCR were utilized to identify PPV in clinical samples to assess the clinical effectiveness of ERA-CRISPR/Cas12a/LFD for detection of PPV. ERA-CRISPR/Cas12a/LFD detection was performed on 15 tissue samples, as shown in **Table 2**. The qPCR analyses revealed that four samples tested positive for PPV. The ERA-CRISPR/Cas12a/LFD results were equivalent to the qPCR results, with a positive rate of 26.7%. These findings demonstrated that ERA-CRISPR/Cas12a/LFD was effective in detecting clinical PPV, and the coincidence rate was 100% when compared with qPCR data. The clinical PPV detection results for the ERA-CRISPR/Cas12a/LFD are shown in **Figure 8**.

**Table 2:** Comparison of qPCR and CRISPR/Cas12a/LFD results for clinical samples.

Assay	Number of samples		Total	Diagnostic accuracy
	Positive	Negative		
CRISPR/Cas12a/LFD Judgment	4	11	15	26.7%
qPCR Result	4	11	15	

qPCR and CRISPR/Cas12a/LFD represent quantitative real-time polymerase chain reaction, reverse-transcription recombinase-aided amplification coupled with lateral flow dipstick, respectively.

## Discussion

PPV can cause reproductive disorders in pigs, most notably infection and death of embryos and fetuses, particularly stillbirth, malformed and mummified fetuses in sows at first farrowing, but sows show no obvious clinical signs. PPV is very insensitive to acid, alkali and heat, and can survive for a long time in the external environment, which seriously affects the litter rate of sows and causes losses to the pig industry (Wu et al., 2014; Streck et al., 2015). As a result, early detection of PPV is critical in clinical practice. The existing PPV detection approach requires specialized equipment and is not suitable for on-the-spot detection. As a consequence, we require a method that does not rely on sophisticated detection apparatus and equipment and can be used for on-site detection in remote places. This procedure should be quick, sensitive, and precise, and it should not necessitate complicated instrumentation.

With the discovery of CRISPR/Cas-based systems, it is possible to develop an accurate, fast and convenient diagnostic test. Multiple pathogens can currently be detected utilizing RPA or ERA in combination with Cas12a, including mycoplasmas, ASFV, PRRSV, and SARS-CoV-2. The test can be completed in less than 30 min, with the results visible under UV light. The technique is extremely sensitive, being capable of detecting a single copy of genomic DNA with a sensitivity that is comparable to or better than that of qPCR (B. Wang et al., 2019; Bai et al., 2019; X. Wang et al., 2020). The sensitivity of a nucleic acid detection approach based on CRISPR-Cas12 is substantially higher than that of traditional PCR, RT-qPCR, RPA, or LAMP. Many detection methods have been reported for PPV infection, including the LAMP and real-time RPA assay (J.-c. Wang et al., 2017; Zhao et al., 2020). The ERA-CRISPR/Cas12a/LFD method developed in this study has higher or equivalent sensitivity to the above methods. The method has no cross-reactivity with other viruses and can specifically detect PPV.

In this study, three pairs of primers were designed for the conserved sequence VP2 of PPV and a rapid detection method of ERA-CRISPR/Cas12a/LFD with a detection limit of 3.75 × 10^2^ copies was established. The testing of clinical samples (liver, spleen and kidney) using ERA-CRISPR/Cas12a/LFD and qPCR showed consistent results between the two approaches, with a positive rate of 26.7% for PPV, demonstrating the practicality and accuracy of the mechanism. The lateral flow dipstick technique was faster, the results were easy to read, and no professional operation was required. The combination of the CRISPR/Cas12a and the lateral flow dipstick technique make this experiment not only rapid, sensitive, and accurate, but also intuitive. This is the first report of PPV detection using CRISPR/Cas12a and LFD, and this approach can be applied to the field detection of PPV in resource-poor areas, which is of high practical value for PPV prevention and control. In summary, this study established a rapid, accurate, simple and intuitive ERA-CRISPR/Cas12a/LFD assay for the specific detection of PPV.

## Data Availability Statement

The original contributions presented in the study are included in the article/supplementary material. Further inquiries can be directed to the corresponding author.

## Ethics Statement

This animal study was reviewed and approved by the Committee on the Ethics of Animal Care and Use at Anhui Agriculture University (Anhui, China). Written informed consent was obtained from the individuals’ and minors’ legal guardians/next of kin, for the publication of any potentially identifiable images or data included in this article.

## Author Contributions

All authors contributed to the article and approved the submitted version. JW, YL and KY designed the research and analyzed the data. XS, YS, KQ and JT provided resources. JW, YL, YC and QL performed the experiments and wrote the manuscript.

## Funding

This work was supported financially by Application of supporting technology and poverty alleviation demonstration of ecological breeding of native black pig in contiguous poverty-stricken area of Dabie Mountains, Anhui (201907d06020016) and 2020 University Excellent Talents Support Program (gxyqZD2020009).

## Conflict of Interest

The authors declare that the research was conducted in the absence of any commercial or financial relationships that could be construed as a potential conflict of interest.

## Publisher’s Note

All claims expressed in this article are solely those of the authors and do not necessarily represent those of their affiliated organizations, or those of the publisher, the editors and the reviewers. Any product that may be evaluated in this article, or claim that may be made by its manufacturer, is not guaranteed or endorsed by the publisher.
